# Sea Buckthorn (*Hippophae rhamnoides*) Waste Biomass after Harvesting as a Source of Valuable Biologically Active Compounds with Nutraceutical and Antibacterial Potential

**DOI:** 10.3390/plants11050642

**Published:** 2022-02-26

**Authors:** Sarmite Janceva, Anna Andersone, Liga Lauberte, Oskars Bikovens, Vizma Nikolajeva, Lilija Jashina, Natalija Zaharova, Galina Telysheva, Maris Senkovs, Gints Rieksts, Anna Ramata-Stunda, Jelena Krasilnikova

**Affiliations:** 1 Laboratory of Lignin Chemistry, Latvian State Institute of Wood Chemistry, Dzerbenes Street 27, LV-1006 Riga, Latvia; sarmite.janceva@kki.lv (S.J.); liga.lauberte@kki.lv (L.L.); oskars.bikovens@kki.lv (O.B.); lilija_jasina@inbox.lv (L.J.); natalija.zaharova@gmail.com (N.Z.); gints.rieksts@inbox.com (G.R.); 2 Ekokompozit Ltd., Dzerbenes Street 27, LV-1006 Riga, Latvia; 3 Faculty of Biology, University of Latvia, Jelgavas Street 1, LV-1004 Riga, Latvia; vizma.nikolajeva@lu.lv (V.N.); maris.senkovs@lu.lv (M.S.); anna.ramata-stunda@lu.lv (A.R.-S.); 4 Department of Biochemistry, Riga Stradiņš University, Dzirciema Street 16, LV-1007 Riga, Latvia; jelena.krasilnikova@rsu.lv

**Keywords:** sea buckthorn twigs, plant secondary metabolites, polyphenols, proanthocyanidins, serotonin, biological activity, antioxidant, antibacterial activity, cytotoxicity, *alpha*-amylase

## Abstract

For sustainable sea buckthorn *(Hippophae rhamnoides)* berry production, the task at hand is to find an application for the large amount of biomass waste arising at harvesting. Sea buckthorn (SBT) vegetation is currently poorly studied. The purpose of this research was to assess the composition and potential of SBT twigs as a source of valuable biologically active substances. Water and 50% EtOH extracts of twigs of three Latvian SBT cultivars with a high berry yield and quality, popular for cultivation in many countries (*H. rhamnoides* ‘Maria Bruvele’, ‘Tatiana’, ‘Botanicheskaya Lubitelskaya’), were investigated for the first time. The phytochemical composition (UHPLC-ESI-MS/MS analysis) and biological activity of the obtained hydrophilic extracts were determined. The highest yield of polyphenolic compounds and serotonin was observed for ‘Maria Bruvele’. Hydrophilic extracts were investigated for radical scavenging activity (DPPH˙ test), antibacterial/antifungal activity against five pathogenic bacteria/yeast, cytotoxicity, and the enzymatic activity of *alpha*-amylase (via in vitro testing), which is extremely important for the treatment of people with underweight, wasting, and malabsorption. The results showed a high potential of sea buckthorn biomass as a source of valuable biologically active compounds for the creation of preparations for the food industry, nutraceuticals, and cosmetics.

## 1. Introduction

SBT (*Hippophae rhamnoides L.*) is a nitrogen-fixing and pest-resistant deciduous shrub tree which grows widely in Europe and high-altitude cold regions of Asia, and North and South America. Surviving the extreme temperatures (from −40 to +40 °C) [1,2] forces the plant to develop adaptogenic qualities. The plant has evolved a diversified chemical portfolio, and every part of the plant is nutritious, including its leaves, twigs, and even bark [3,4].

The global SBT berries export market is valued at USD 2 billion. The top exporter in 2020 was Canada, with USD 0.417 billion and a yearly increase of 13.9% [5]. On an industrial scale, SBT is cultivated in Russia, China, Canada, Finland, Germany, Latvia, Romania, and Estonia [6]. The overall market of SBT products is ~17 times bigger than just for berries and is constantly growing [7]. The industry uses fruits, but there are almost no applications for the green part of SBT [5], which comprises ~12–15% of the harvested mass [8,9].

Varietal characteristics have a significant impact on the quality indicators of raw material [10,11,12]. The advantages of Latvian SBT varieties are frost resistance, high-quality (large and sweet) fruits, and winter hardiness. Among the varieties exported to different countries, the largest-fruited (0.5–1 g) are *H. rhamnoides* ‘Botanicheskaya Lubitelskaja’, ‘Prozrachnaya’, ‘Maria Bruvele’, ‘Tatiana’, ‘Avgustinka’, ‘Perchik’, and ‘Trofimovskaya’ [13,14,15] (henceforth to be identified as the cultivars ‘Botanicheskaya Lubitelskaja’, ‘Prozrachnaya’, ‘Maria Bruvele’, ‘Tatiana’, ‘Avgustinka’, ‘Perchik’, and ‘Trofimovskaya’).

SBT fruits are quite well studied in terms of phytochemical composition and application [16,17,18,19,20]. The application of SBT fruit oil and leaf has no side effects [21]. The demand for natural biologically active substances, including antioxidant, antibacterial, and biostimulating substances, replacing harmful chemically synthesized ones, is constantly growing. Plant secondary metabolites possess enormous potential for further uses [22].

Upon harvesting at the production scale, a large amount of SBT biomass waste is produced, as the berries are collected by cutting the whole branch, freezing it, and shaking off the frozen berries. When the plant rests from growing berries, pruning is carried out to rejuvenate the bushes. Finding applications for the SBT biomass waste is necessary for the sustainable use of resources, which is the task of the European Green Deal [1], and for the creation of additional income for SBT growers and workers in rural area.

Sea buckthorn (SBT) is one of the most ancient plants on Earth (older than 2 billion years), and its fruits are mentioned among the most valuable in the world [8,23,24]. In recent studies, it was found that the biomass of all parts of the SBT tree also contains practically all biologically active groups of organic compounds currently known and 18 important microelements [1,25]. This is why in Latvia SBT is called the Latvian Gold. This makes the biomass of SBT a promising raw material for different branches of the economy [26].

SBT accumulates significant amounts of polyphenolic substances, including flavonols, flavones, phenolic acids, proanthocyanidins (PACs), and hydrolysable tannins, which are reported as the major contributors to antioxidant activities of SBT berries and leaves [17,27], and could be used both for the creation of pharmaceuticals and in the food industry to slow down oxidative processes in raw materials and finished products.

SBT bark contains another valuable secondary metabolite, serotonin [28], which is one of the most interesting and expensive components of SBT extractives. The research on obtaining serotonin from SBT is very limited worldwide.

**Figure 1:**
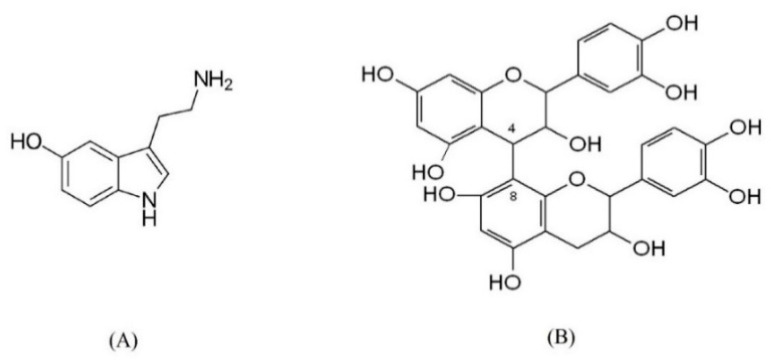
Chemical structure of serotonin (**A**) and B-type procyanidin (**B**), represented by 4–8 epicatechin dimer.

The chemical structures of serotonin and B-type procyanidin are shown in Figure 1.

The levels of serotonin vary in different plant parts [29]. Serotonin has been implicated in diverse physiological functions in plants such as growth regulation, flowering, xylem sap exudation, ion permeability, plant morphogenesis, and the regulation of abiotic stress tolerance [30]. It also defends plants against fungi [31]. In mammals, serotonin acts as a neurotransmitter in the central nervous system and affects motor activity and the functioning of the gastrointestinal tract. It can be beneficial for treating cancer, HIV, Parkinson-like symptoms, obesity, depression, insomnia, alcohol abuse, schizophrenia, and several chronic diseases [28,32]. The content of serotonin in the bark of SBT is one-thousand times higher than in bananas or chocolate [28]. The antioxidant activity of serotonin far exceeds that of tryptophan, tryptamine, and serotonin derivatives [33]. Furthermore, 95% of serotonin is produced in the peripheral organs, and serotonin in the digestive system may work independently of serotonin in the brain [34]. In addition to its application in human diets, serotonin could be used as a natural biostimulant for plant rooting [30].

Another important urgent task is the search for natural substances with antibacterial properties and low toxicity to the human body since the resistance to synthetic antibiotics among Gram-positive and Gram-negative bacterial pathogens is growing tremendously. Each year, approximately 25,000 patients in the EU die from infections due to multidrug-resistant bacteria [35]. *Escherichia coli* and *Staphylococcus aureus* are pathogens that are responsible for the most primary and secondary skin and blood infections. *S. aureus* also constitutes 30% of burn wounds. Non-healing wounds are a huge problem for diabetic and older patients [36]. Extracts of the vegetative part of SBT have not yet been studied as antimicrobial agents, although studies on the effect of PACs and quinic acid on the activity of *E. coli* in cells indicate the possibility of creating antimicrobial agents based on them [37]. Quinic acid inhibits the growth of most microorganisms [38].

An equally important problem is metabolic disorders in the body. There is evidence of the effect of PACs on the activity of the hydrolytic cleavage of carbohydrates to monosaccharides, under the action of α-amylase in saliva [39,40]. It has been proven that the initial processes of human macro-metabolism are extremely important, and saliva plays a major physiologic role in food digestion [41]. Studies have linked higher amylase levels to better glucose tolerance after eating starch-rich meals [42,43]. PAC-containing extracts, based on the concentration of the active substance, can both activate and inhibit the activity of amylase in the initial breakdown of carbohydrates. Therefore, the possibility of using SBT twig extracts for the normalization of the enzymatic activity of saliva can be studied.

Taking into account the relevance of the topic of the complex and rational use of plant raw materials, and the insufficient amount of knowledge about SBT biomass, the aim of this study was to assess the potential of pruned SBT twigs, as waste after SBT berries harvesting, of three the most prospective cultivars of SBT (‘Maria Bruvele’, ‘Tatiana’, and ‘Botanicheskaya Lubitelskaya’) as a source of valuable biologically active substances, mainly polyphenols and serotonin, with the establishment of the phytochemical composition and biological activity (antioxidant, antibacterial, and enzymatic) of the obtained hydrophilic extracts to determine their practical significance.

## 2. Results and Discussion

### 2.1. Yield and Chemical Composition of Hydrophilic Extracts

**Figure 2:**
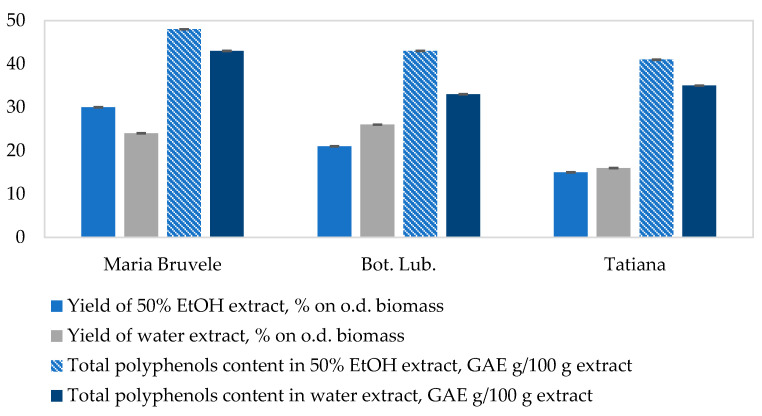
Effect of the extractants on the extract yield from twigs of SBT and selectivity for polyphenolic compounds (single-step extraction, 30 min, 60 °C, biomass and extractant weight ratio 1:8). Data represented as mean ± SD (*n* = 3).

A review of the published literature data [44] and our preliminary experiments [45,46] showed that ethanol (EtOH) and its aqueous solutions are the most suitable extraction solvents for the isolation of biologically active polyphenolic compounds from plant biomass, including SBT biomass. For the study of three SBT cultivars (‘Maria Bruvele’; ‘Tatiana’; and ‘Botanicheskaya Lubitelskaya’—‘Bot. Lub.’), 50% EtOH and distilled water were used as extractants for hydrophilic extract isolation, based on their selectivity for polyphenolic compounds, chemical inertness, low toxicity, and low cost. The quantitative analysis of the total polyphenolic compounds was performed for the obtained extracts, determining the effect of extractant concentration on the efficiency of polyphenolic compound release (Figure 1). The yield of hydrophilic extracts was determined by the gravimetric method after the freeze-drying of the samples. The yield of hydrophilic extracts (hereinafter extracts) from twigs of three SBT cultivars varied from 15% to 30% in terms of dry SBT biomass (Figure 1). The obtained extracts were rich in polyphenolic compounds that are known to be responsible for free radical scavenging activity. The ‘Maria Bruvele’ biomass had the highest yield of 50% EtOH extract (30% from o.d. biomass) and the highest content of phenolic compounds in the extracts (48.1 GAE g/100 g extract or 14.4% on o.d. biomass with confidence interval CI = 0.2 at α = 0.05). Despite the high content of the total polyphenols in 50% EtOH and water extracts of ‘Tatiana’ (41.3 and 35.1 GAE g/100 g extract, CI = 0.2 at α = 0.05), the yield of the extracts themselves, compared to ‘Maria Bruvele’, was 2 times less (15 and 16% on o.d. extract, with CI = 0.4 at α = 0.05), which reduced the yield of polyphenols from SBT biomass (6.2% and 5.6% on o.d. biomass). Based on these observations, ‘Maria Bruvele’ is the raw material with the most potential between the 3 investigated popular cultivars for obtaining polyphenol-rich extracts, which has also been confirmed by other authors by examining the chemical composition of sea buckthorn twigs grown in Poland [16]. The contents of total polyphenolic compounds of all the extracts are given in Figure 2.

**Figure 3:**
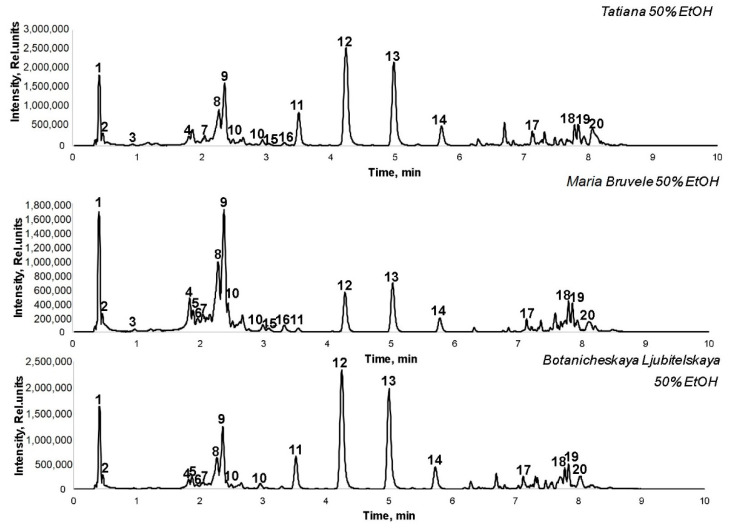
UHPLC-TOF/MS chromatograms of 50% EtOH extracts of SBT samples.

**Figure 4:**
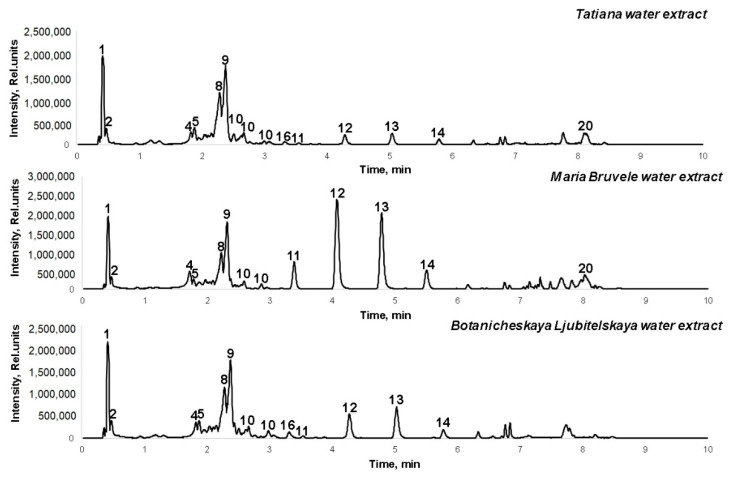
UHPLC-TOF/MS chromatograms of the water extracts of SBT samples.

The freeze-dried 50% EtOH and water extracts were analyzed by UHPLC-ESI-MS/MS for the identification of biologically active compounds, including polyphenolic compounds, representing a significant part of the extract. UHPLC-ELS chromatograms of extracts are shown in Figure 3 and Figure 4.

The compounds identified are listed in Table 1, with the most abundant ones being quinic acid, catechin/epicatechin, gallocatechin, procyanidin trimer, procyanidin tetramer, (epi)catechin-(epi)gallocatechin, quercetin, quercetin-3-O-rutinoside, triterpenoids, and acylated triterpenoids. Part of the polyphenolic compounds in the extracts is in the form of O-glycosides, which consist of a residue of aglycone and carbohydrates consisting mainly of glucose. At the same time, the compositional similarity of the composition of the 50% EtOH and water extracts of the branches of all three varieties of sea buckthorn was found. All these compounds are biologically active natural antioxidants and antimicrobial agents, which can be used as ingredients in the formulation of different medications.

Among the identified polyphenolics compounds, quinic acid, quercetin, and triterpenoids have documented excellent antibacterial activity against *Staphylococcus aureus*, which is a leading Gram-positive pathogen associated with a number of diseases, including osteomyelitis, pneumonia, endocarditis, and septicemia. This bacterium is also frequently found in many food products such as dairy, eggs, seafood, and meat, and can cause food poisoning, which is a major concern for the international community and the food industry. Additionally, they show antibacterial activity toward Staphylococcus epidermidis, Bacillus subtilis, and Escherichia coli [37].

A comparison of the relative peak areas of the dominant biologically active compounds calculated for mg/mg extract is shown in Table 2.

### 2.2. Evaluation of the Antimicrobial Activity of SBT Extracts

The evaluation of the antimicrobial activity of the plant extracts against test cultures of microorganisms was carried out according to the method for determining the sensitivity of microorganisms to antimicrobial drugs. Antimicrobial activity was studied in 96-well plates by the two-fold serial broth microdilution method, which allowed the determination of the minimum inhibitory (MIC) and minimum bactericidal/fungicidal concentrations (MBC/MFC) [47].

The comparison of the results of the chemical composition and antimicrobial activity of 50% EtOH extracts showed that among the three varieties of SBT, ‘Maria Bruvele’ was the most active in suppressing pathogenic bacteria. This could be explained by the increased content of such biologically active compounds as quinic acid, gallocatechin, isomer epigallocatechin, (epi)catechin-(epi)gallocatechin, and procyanidins.

The results show that all extracts have high antimicrobial activity against both Gram-positive and Gram-negative bacteria (see Table 3).

When comparing the 50% EtOH and water extract of SBT ‘Maria Bruvele’, we found that despite the fact that the content of quinic acid in the water extract was 1.24 times higher, the 50% EtOH extract was more active; this could be attributed to the synergetic activity with the other antimicrobial agents, namely, an increased amount of such polyphenolic compounds as gallocatechin or isomer epigallocatechin (1.25 times higher in the 50% EtOH extract), (epi)catechin-(epi)gallocatechin (1.13 times higher) and procyanidins (2.5 times higher).

The minimum bactericidal concentration of the extracts showed that the extracts were able to completely neutralize the bacteria under study, and their MICs were even comparable with those of weaker antibiotics.

### 2.3. Evaluation of the Cytotoxicity of Extracts

**Figure 5:**
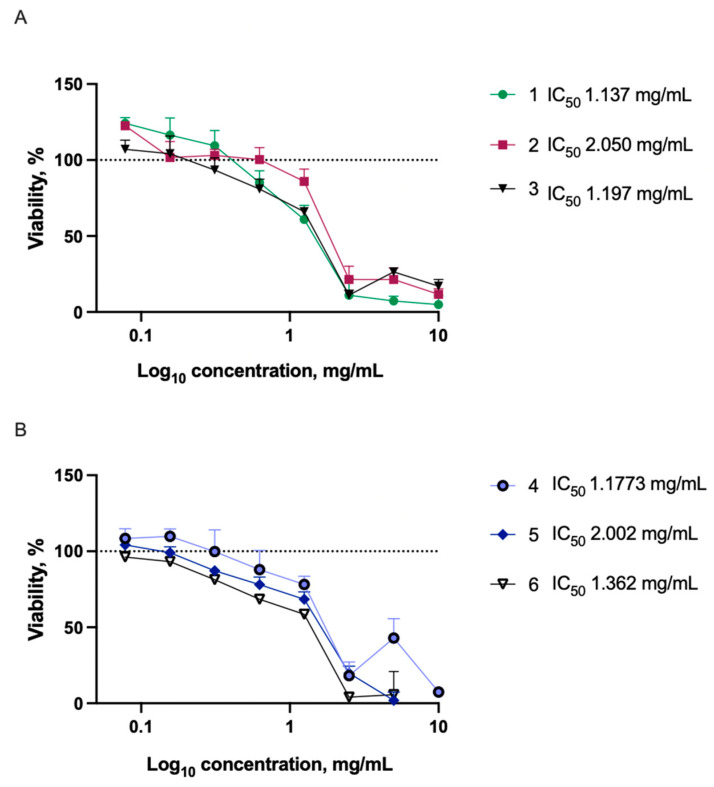
Cytotoxicity of extracts in Balb/c 3T3 cell line. Results expressed as a relative change compared to untreated control. (**A**)—50% EtOH extracts: 1—Maria Bruvele; 2—Tatiana; 3—Bot. Lub.; (**B**)—water extracts: 4—Tatiana; 5—Bot. Lub.; 6—Maria Bruvele. Data represented as mean ± SD (*n* = 3). Dotted line represents the control level (100%).

The graphs below show the results of the cytotoxicity tests, by changes in cell viability and IC_50_ values. It was reasonable to evaluate the cytotoxicity around the range of the antimicrobial activity of the extracts. The testing started from lower concentrations of 0.078 mg/mL up to 10 mg/mL (Figure 5). The use of higher concentrations made it difficult to read the results objectively as the intensive coloring influenced the absorbance.

In most cases, at concentrations similar to the MIC values observed in antimicrobial activity tests, no toxic effect was observed. Water extracts were slightly more cytotoxic than ethanol extracts. An extract at a specific concentration was considered to be cytotoxic if the cell viability was reduced by more than 20%. Cytotoxic concentrations of ethanol extracts (‘Maria Bruvele’, ‘Tatiana’) did not exceed the concentrations needed to inhibit the growth of the tested microorganisms. Some variations in the effects of water extracts on cell viability were observed, with the ’Tatiana’ extract being less cytotoxic than the other two water extracts. Compared to other studies, extracts tested here have low cytotoxicity. Triterpenoid-rich SBT extracts have been reported to be cytotoxic to cancerous and normal human cell lines at lower concentrations than in our study, with IC_50_ values ranging between 14.58–74.58 μg/mL [48]. SBT extract within concentration range 0.62–62 μg/mL was shown to have no negative effects on NIH 3T3 cell line but reduced viability of glioma cells in vitro. This emphasizes the variations of the effects in different cell lines [49]. At the same time, in a study by Rozalska et al., IC_50_ values above 1 mg/mL for phenolic SBT fractions have been reported [50]. The effects on cell lines depend on the extract production methods and chemical composition. Varying cytotoxicity data among studies might be explained mainly by the variations in chemical composition on SBT extracts.

The low concentrations needed to inhibit the growth of specific microorganisms, especially *C. albicans* and *S. aureus*, along with the absence of cytotoxicity at low concentrations, indicate on the potential of the tested extracts to be further developed for various antimicrobial applications. The low concentrations needed to inhibit the growth of specific microorganisms, especially *C. albicans* and *S. aureus*, along with the absence of cytotoxicity at low concentrations, indicate to the potential of the tested extracts to be further developed for various antimicrobial applications.

Interestingly, at low concentrations, ethanol extracts of ‘Maria Bruvele’ and ‘Tatiana’ slightly increased cell viability and proliferation (increases of 24.13 and 22.59%, respectively). This phenomenon could be further researched in future studies.

### 2.4. The Radical Scavenging Activity of SBT Extracts

**Figure 6:**
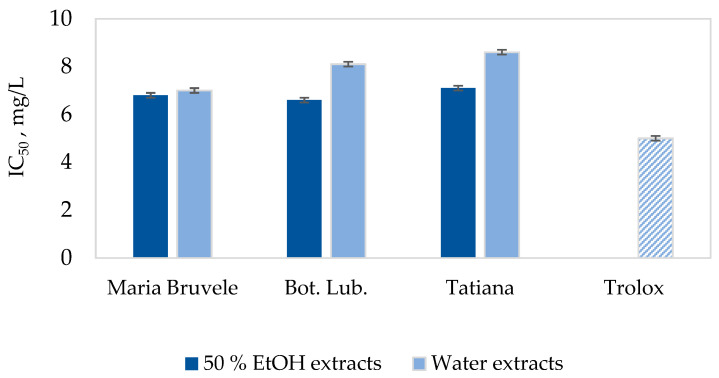
Radical scavenging activity of SBT biomass extracts by DPPH˙ test. Data represented as mean ± SD (*n* = 3).

The radical scavenging activity of SBT extracts was evaluated by DPPH˙ tests, expressed as the IC_50_ value, the concentration required for the 50% inhibition of free radicals [51]. The results of the radical scavenging activity assays are shown in Figure 6. Among the SBT samples under study, the 50% EtOH extracts of ‘Maria Bruvele’ and ‘Bot. Lub.’ twigs manifested the highest radical scavenging activity (IC_50_ = 6.8 mg/L and 6.6 mg/L) compared to the other extracts.

**Figure 7:**
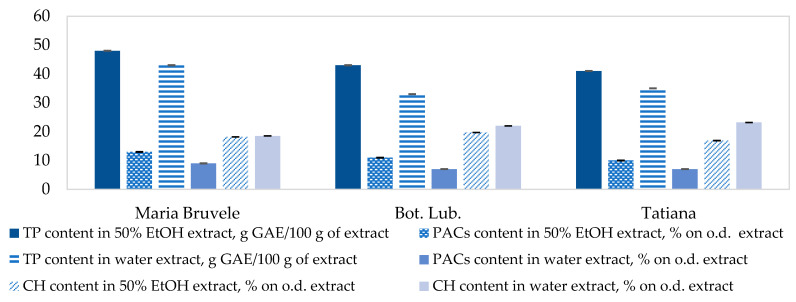
Chemical composition of extracts from SBT biomass. Data represented as mean ± SD (*n* = 3).

As shown in Figure 7, such high activity of these extracts is associated with a high content of polyphenolic compounds (48 and 43 GAE g/100 g extract), including PACs (13 and 11% on the o.d. extract, CI = 0.2 at α = 0.05).

**Figure 8:**
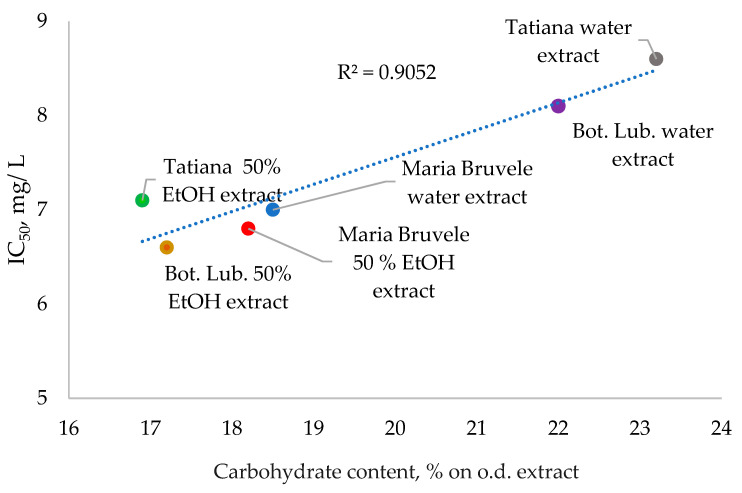
Effect of carbohydrate content on radical scavenging activity. Confidence intervals for antioxidant activity: CI ≤ 0.1 at α = 0.05, for carbohydrate content: CI ≤ 0.03 at α = 0.05.

When comparing the radical scavenging activity of the extracts from SBT twigs, a correlation between the content of carbohydrates as unwanted compounds in the extracts and their radical scavenging activity could be observed (Figure 8). With the increase in carbohydrate content in the extracts, their radical scavenging activity decreased in the DPPH˙ test. The water extract of Tatjana twigs had a weaker ability to deactivate radicals due to the high content of carbohydrate impurities (23% on o.d. extract, CI = 0.03 at α = 0.05) and low content of PACs (7% on o.d. extract) in the extract. The polar groups of carbohydrate impurities can form hydrogen bonds with the hydroxyl groups of polyphenolic compounds, thus reducing their radical scavenging activity. The carbohydrate content in the 50% EtOH and water extracts of SBT biomass is shown in Figure 8.

**Figure 9:**
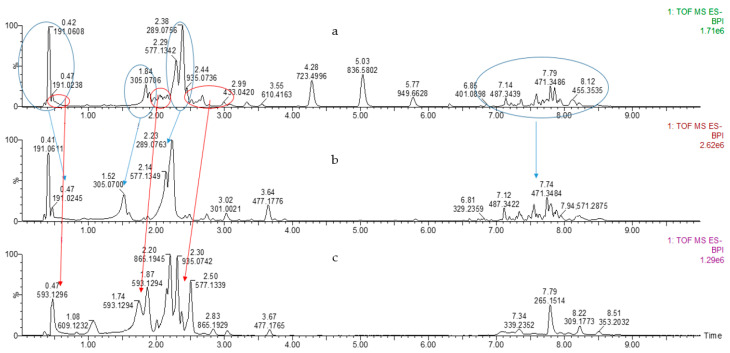
UHPLC-ELS chromatograms: (**a**)—50% EtOH extract of ‘Maria Bruvele’ biomass; (**b**)—low molecular compound rich fraction; (**c**)—procyanidin trimer rich fraction.

One way to increase antioxidant activity is to remove impurities from the dominant components with strong antioxidant properties. Our preliminary research indicates that proanthocyanidins are powerful antioxidants. Based on this, it was decided to purify proanthocyanidins by Sephadex LH-20. As a result, two fractions were obtained, which were also characterized by UHPLC-ELS (Figure 9).

In comparison to the synthetic antioxidant Trolox as a reference, which is a water-soluble derivative of vitamin E (IC_50_ = 5 mg/L in DPPH˙ test), the 50% EtOH extract of ‘Bot. Lub.’ and ‘Maria Bruvele’ showed the most promising results (IC_50_ = 7.1 mg/L in DPPH˙ test, CI = 0.1 at α = 0.05). The antioxidant activity of purified procyanidin was significantly higher (IC_50_ = 2.6 mg/L in DPPH˙ test, CI = 0.1 at α = 0.05).

### 2.5. The Content of Serotonin in SBT Extracts

**Figure 10:**
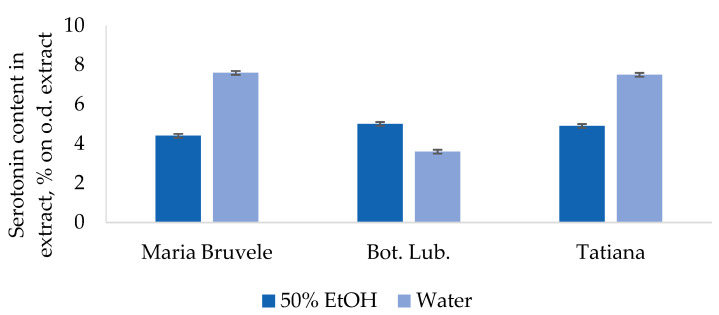
Content of serotonin in extracts from SBT biomass. Data presented as mean ± SD (*n* = 3). CI ≤ 0.04 at α = 0.05.

Of the alkaloid compounds, SBT lignocellulosic biomass contains serotonin, which is a powerful antidepressant and stimulant of psychological and physical activity. The analysis of liquid chromatography (UHPLC-ELS) proved its presence in the composition of hydrophilic extracts. The serotonin content in the SBT extracts (Figure 10) varied from 3.6 to 7.5% per dry extract, and, starting from the highest content, decreased in the following order: water extracts of ‘Maria Bruvele’ and ‘Tatiana’ >50% EtOH extract of ‘Bot. Lub.’ >50% EtOH extract of ‘Maria Bruvele’ > water extract of ‘Bot. Lub.’.

When using extracts as biologically active food additives or as an additional component of activators of food enzymes, serotonin will only increase the value of this biologically active product.

To prove the potential of the extract’s biological activity, in the following stage, in vitro experiments were performed.

### 2.6. Evaluation of the Influence of the Extracts on Amylase Activity

In vitro tests were also carried out for the evaluation of the influence of the extracts on the initial processes of human macro-metabolism, which is relevant for the extract usage for health care and disease prevention. Earlier tests in collaboration with Riga Stradinš University in this regard have revealed the beneficial effects of PACs-containing extracts on amylase activity, resulting in the acceleration of starch degradation to glucose, which could be useful for the treatment of persons with underweight, malnutrition, and malabsorption [52,53]. Under normal physiological conditions, all extracts at dosages of 100 µL, 500 µL, and 1000 µL at an extract concentration of 2 mg/L showed a significant activation (two times) of amyloclastic force (AF). With the increase in the extract concentration from 2 mg/L to 20 mg/L, at dosages of 100 µL and 500 µL of the extracts, the same activation was observed. Increased α-amylase activation can accelerate the degradation of starch to glucose, which may be useful in the treatment of people with underweight, malnutrition, and malabsorption.

## 3. Materials and Methods

### 3.1. Materials

The twigs (without leaves) of three sea buckthorn cultivars—*H. rhamnoides* ‘Maria Bruvele’, ‘Botanicheskaya Lubitelskaya’, and ‘Tatiana’—were collected from the sea buckthorn plantation area in Latvia under the same growing conditions in summer of 2020. Varieties suitable for commercial growth in the NE of Europe and Canada, larger and more juicy fruits with better taste, and fruits with significantly less troublesome stellate hairs were chosen. The twigs were dried at room temperature, ground with a knife mill (Cutting Mill SM100, Retsch, Haan, Germany) and sieved to select the particles between 1 and 4 mm. These fractions were stored at −8 °C.

### 3.2. Isolation of the Hydrophilic Extracts from Twigs of Sea Buckthorn Biomass

Hydrophilic extracts were isolated by the convective extraction of SBT biomass at 60 °C for 30 min using the following solvents: distilled water or aqueous ethanol (1:1, *v/v*). The extracts were freeze-dried to yield a brown solid. The yield of the extracts is presented as a percentage based on the oven-dried (o.d.) biomass.

### 3.3. Total Polyphenols Content in the Extracts

The total polyphenols (TP) content of the extracts was determined using the Folin–Chicolteu method using gallic acid as the standard. Amounts of 5 mL of 10% Folin–Ciocalteu reagent and 4 mL of 7.5% sodium carbonate solution were added to 1 mL of the extract. Distilled water was used instead of gallic acid as a reference solution. After 30 min, the absorbance of the mixture was measured against a blank solution at 765 nm using a UV/VIS spectrometer Lambda 650 (Perkin Elmer, Shelton, CT, USA). Gallic acid was used to calibrate the standard curve. Each extract was analyzed in triplicate, and the results were expressed in grams of gallic acid per 100 g of extract sample (g GAE/100 g extract) [54].

### 3.4. Proanthocyanidins Content in the Extracts

The PACs content of the extracts was determined by oxidative depolymerization to anthocyanidins in acid butanol [55] using procyanidin dimer B2 as a reference compound. Amounts of 6 mL of acid butanol (5% (*v/v*) concentrated HCl in n-butanol) and 0.2 mL of iron reagent (*w/v*) (FeNH_4_(SO_4_)_2_∙12 H_2_O in 2 M HCl) were added to 1 mL of the extract aliquots while stirring the tube without heating and allowing it to be heated in a water bath at 80 °C for 50 min. After 50 min, the absorbance of the mixture was measured against a blank solution at 550 nm using UV/VIS spectrometer Lambda 650 (Perkin Elmer, Shelton, CT, USA). Each extract was analyzed in triplicate, and assay results were expressed as a percentage per o.d. extract.

### 3.5. Purification of PACs

The purification of PACs from non-tannin phenolics and sugar was carried out using a Sephadex LH-20 with 96% EtOH and 70% (*v/v*) acetone as the respective purification solvents. In the purification process, low-molecular-weight phenolics were eluted with 96% EtOH, and the PACs were eluted with 70% (*v/v*) acetone. Purified PACs were evaporated using a rotary evaporator prior to being freeze-dried and stored at −8 °C.

### 3.6. Carbohydrate Content in the Extract

The total amounts of the carbohydrate in the extracts were determined using GC analysis after hydrolysis, reduction, and acetylation [56]. Extract hydrolysis: 0.125 mL of sulfuric acid was added to 10 mg of the extract; after 45 min, it was diluted with 3.5 mL of water and placed into a thermostat for 1 h at 121 °C. After hydrolysis, the sample was neutralized with 0.32 mL of ammonium hydroxide solution and 0.1 mL of analytical standard methyl α-D-glucose. Reduction and acetylation: 1 mL of borohydride solution was added to 0.2 mL of neutralized solution and heated for 90 min at 40 °C. The excess reagent was partitioned with 0.1 mL of concentrated acetic acid. After reduction, 2 mL of acetic anhydride and 0.3 mL of 1-methylimidazole were added. After 10 min (30 °C), the excess acetic anhydride was partitioned with 5 mL of distilled water. The cooled solution was extracted once with 1 mL of CH_2_Cl_2_. The lower layer was transferred to the chromatography flask with a Pasteur pipette and stored at −20 °C until gas chromatographic analysis. Gas chromatographic analysis was performed using an Agilent 6850 Series GS System (Agilent Technologies, Santa Clara, CA, USA): column—DB-1701; length—30 m; internal diameter—0.25 mm; layer thickness—0.25 µm.

### 3.7. UHPLC-ESI-MS/MS Analysis

Extract analysis was performed on an Acquity UPLC system (Waters Corp., Singapore) coupled with a quadrupole-time of flight (Q-TOF) MS instrument (UPLC/SYNAPT G2Si HDMS Q-TOF Mass Spectrometer, Waters, Milford, MA, USA) with an electrospray ionization (ESI) source.

The separation was carried out on a U-HPLC column (2.1 mm × 50 mm i.d., 1.7 µm, BEHC18) (Waters Acquity) at a flow rate 0.35 mL/min. The eluent was 0.1% formic acid, water (A), and acetonitrile (B). A gradient solvent system was used: 0–1 min, 5–20% (B); 1–5 min, 20–25% (B); 5–6 min, 25–75% (B), 6–7 min, 75–80% (B), 7–8 min, 80–5% (B), 8–10 min, 5% (B). The injection volume was 2.0 μL.

The major operating parameters for the Q-TOF MS were set as follows: capillary voltage, 2.5 kV (-); cone voltage, 60 V; cone gas flow, 50 L/h; collision energy, 6 eV; source temperature, 120 °C; desolvation temperature, 350 °C; collision gas, argon; desolvation gas, nitrogen; flow rate, 500 L/h; data acquisition range, m/z 50–1.200 Da; ionization mode negative.

### 3.8. Determination of the Antimicrobial Activity

The antimicrobial activity tests of the hydrophilic extracts from the sea buckthorn twigs were performed at the Faculty of Biology, University of Latvia. To determine antimicrobial activity, several reference microbial strains, received from the Microbial Strain Collection of Latvia (MSCL), University of Latvia, were used: *Pseudomonas aeruginosa* MSCL 334, *Staphylococcus aureus* MSCL 330, *Escherichia coli* MSCL 332, *Bacillus cereus* MSCL 330, and *Candida albicans* MSCL 378. The evaluation of the antimicrobial activity of the plant extracts against the test cultures of microorganisms was carried out according to the method for determining the sensitivity of microorganisms to antimicrobial drugs. Antimicrobial activity was studied in 96-well plates by the two-fold serial broth microdilution method [49], which allowed the determination of the minimum inhibitory (MIC) and minimum bactericidal/fungicidal concentrations (MBC/MFC). The MIC was determined as the lowest concentration of the studied material, which showed no visible growth. From wells where growth was not detected, 4 μL of medium was seeded on an appropriate solidified medium for MBC/MFC determination. The test was performed in triplicate.

### 3.9. Cell Line and Cultivation

The BALB/c 3T3 murine fibroblast cell line was obtained from ATCC (American Type Culture Collection). Cells were propagated in DMEM medium (Sigma, D6046, Irvine, UK) supplemented with 1% penicillin (100 U/mL)–streptomycin (100 μg/mL) and 10% calf serum (Sigma, C8056, St Louis, MO, USA). All cultivations were performed in a humidified 5% CO_2_ atmosphere at 37 °C.

### 3.10. Cytotoxicity Assay

The cytotoxicity of the extracts was tested for the BALB/c3T3 cell line by the neutral red (NR) uptake assay. Cells were seeded in 96-well plates at a density of 5 × 10^3^ cells per well. After 24 h of incubation, extracts in a concentration range of 0.078 to 10 mg/mL were added. Dilutions were made in a cell cultivation medium. Cultivation in the presence of extracts was performed for 48 h. Afterwards, the plates were washed with phosphate-buffered saline (PBS) (Sigma, D8537, Irvine, UK), and 25 µg/mL NR solution (Sigma, N2889, Irvine, UK) diluted in 5% fetal calf serum containing media was added. After 3 h incubation in a humidified 5% CO_2_ atmosphere at 37 °C, the plate was washed with PBS, and the NR taken up by viable cells was extracted using desorbing fixative (50% ethanol/1% acetic acid/49% water). Absorbance at 540 nm was measured using a Tecan M200 Infinite Pro microplate reader (Tecan, Switzerland). Cytotoxicity was expressed as a concentration-dependent reduction in the uptake of NR, compared to the untreated controls, and the IC_50_ value for each compound was calculated using GraphPad 9 software. The cell line and test method complied with OECD guidelines [57].

### 3.11. Determination of the Radical Scavenging Activity

Hydrophilic extracts were tested for their radical scavenging activity against the DPPH˙ assay [51] using UV/VIS spectrometer Lambda 650 (Perkin Elmer, Shelton, CT, USA). The free radical scavenging activity is expressed as the concentration of antioxidant, mg/L, required for a 50% inhibition of the free radicals (IC_50_). The lower the IC_50_ value, the higher the radical scavenging activity of the compounds.

### 3.12. In Vitro Test of the Alpha-Amylase Activity

In vitro tests of the hydrophilic extracts from sea buckthorn samples were performed at the Department of Human Physiology and Biochemistry of Riga Stradinš University based on the determination of amyloclastic force by starch-iodine color assay [53,54]. The saliva used for research was donated on a volunteer basis by a group of students with no record of chronic or acute illnesses. The students were non-smokers, as smoking can increase amylase activity [58]. Any sporting or other serious physical activity was stopped 48 h before the experiment according to the protocol [59]. No chewing gum was allowed. No alcohol or caffeine was taken 18 h before the experiment, and the last meals and soft drinks with low pH were 2 h before the examination to obtain clean results.

The extracts were tested in the doses from 100 µL to 500 µL at a concentration of extracts from 2 mg/L to 20 mg/L. The influence of the extracts on salivary amylase was measured by the breakdown of polysaccharides containing linear α-1,4 glucose bonds in starch. The amylase activity was characterized by the amyloclastic force (AF), that is, the volume of the 0.1% starch solution in milliliters that is hydrolyzed by 1 mL of saliva in the test tubes at 38 °C for 30 min. Then, 1% iodine solution was added (as a marker for the presence of starch by color changes). The amyloclastic force is denoted as D _30/38°_. The average range of AF for a healthy person is from 320 to 1280. Saliva without extract was used as a reference. The amyloclastic force of the reference sample was D _30/38°_ 640.

### 3.13. Statistical Analysis

All measurements were conducted in triplicate, and the results are presented as the mean value ± standard deviation (SD). Statistical analyses were performed using Microsoft Excel 2016. Confidence intervals for a mean using a Student’s T distribution were calculated at a significance level α = 0.05. The correlation is presented by the Pearson coefficient. A significance level of *p* < 0.05 was used.

## 4. Conclusions

The chemical composition of the water and ethanol extracts from the SBT biomass of three prospective cultivars, *H. rhamnoides* ‘Maria Bruvele’, ‘Tatiana’, and ‘Botanicheskaya Lubitelskaya’, was determined for the first time. The results of UHPLC-ELS chromatograms showed that the extracts of SBT are rich in polyphenolic compounds, such as quinic acid, PAC monomers and oligomers, which predetermines their radical scavenging activity. Maria Bruvele twigs biomass has the highest content of phenolic compounds in the 50% EtOH extract (48 GAE g/100 g extract).

The 50% EtOH extracts of ‘Maria Bruvele’ and ‘Botanicheskaya Lubitelskaya’ twigs showed the most promising results for radical scavenging activity (IC_50_ = 6.8 mg/L and 6.6 mg/L, in the DPPH˙ test) compared to the other extracts. This could be connected to the higher content of polyphenolic compounds (48 and 43 GAE g/100 g extract), including PACs (13 and 11% on the o.d. extract). These extract properties can be valuable not only in the creation of pharmaceuticals on the basis of plant secondary metabolites, but also in the food and cosmetics industries to slow down the oxidative processes occurring in raw materials and finished products at different stages of the technological process during storage.

Among the three varieties of SBT, ‘Maria Bruvele’ 50% EtOH extract’s antimicrobial activity in suppressing the pathogenic bacteria and yeast is the highest, and its effectiveness against bacteria is comparable with that of some of the synthetic antibiotics. This could be explained by the high content of quinic acid, gallocatechin, isomer epigallocatechin, (epi)catechin-(epi)gallocatechin, and proanthocyanidins in the ‘Maria Bruvele’ extract and their possible synergetic activity. These results provide the scope of further research on the SBT extracts for applications in the prevention and treatment of infectious diseases. At concentrations similar to the MIC values observed in antimicrobial activity tests, no toxic effect was observed, thus confirming the potential for practical use of the extracts.

The serotonin content in the SBT extracts varied from 3.6 to 7.5% per dry extract, and, starting from the highest content, decreased in the following order: water extracts of ‘Maria Bruvele’ and ‘Tatiana’ >50% EtOH extract of ‘Bot. Lub.’ >50% EtOH extract of ‘Maria Bruvele’ > water extract of ‘Bot. Lub.’. As the serotonin content of 4–6% per dry extract is already considered in the literature as high, these results are very promising for obtaining serotonin preparations on the basis of SBT. The discovery of mammalian neurohormones in plants provides new avenues for the investigation of medicinally active compounds. There is a lack of research on the effects of serotonin. As SBT is a very stress-resistant plant, it would be very interesting to explore if serotonin additives would help to improve similar characteristics in the human body. Currently, the concept of the influence of the gut microbiota on the central nervous system is gaining ground and building evidence. In this case, serotonin in the digestive system could affect mood and brain health. Further research on serotonin effects is planned.

In vitro tests carried out for the evaluation of the influence of the extracts on the initial processes of human macro-metabolism, presented by saliva amylase activity, showed that under normal physiological conditions, all extracts have a significant activation (two times higher than the reference) of amyloclastic force (AF). These results are very promising for the treatment of people with underweight, malnutrition, and malabsorption. The effect of combined SBT extracts on amylase activation and serotonin anti-depression and physical activity improvement could also be beneficial.

The results of this research showed that the biomass of the cultivars under study has high biological activity and nutraceutical and pharmaceutical potential, and the creation of a wasteless biorefinery processing scheme for SBT sustainable production including both berries and biomass applications is possible and prospective. SBT acreage is evaluated as 3 mln ha in 2017 [8], and average crop productivity is ~4–5 t/ha [1,8,9], that is, potentially 13,5 mln t annually. Considering that biomass volume, as mentioned above, comprises ~12–15% of berries, an average volume of biomass could be ~2 mln t yearly. The potential contribution of twigs to the market values will depend on the ways of application: as a raw biomass, as an extract, as a group of compounds, or as purified compounds. On the example of PACs, considering the average for all cultivars yield of extracts of 23%, average yield of PACs 10% on o.d. biomass, and the very approximate price for raw material 10 EUR/kg [60], the market value for PACs could be very roughly estimated as EUR 480 million annually. However, if to calculate on the basis of the final product packed as biologically active additives and sold, e.g., on iHerb [61] then the price could reach 5700 EUR/kg, and the market value theoretically could increase up to EUR 260 billion annually. That would be a hardly achievable figure considering the worldwide market value, the global health and wellness market is forecast to be worth one-trillion dollars by 2026 [62]. The theoretical figures would increase even more if we take, for example, analytical standard serotonin as a basis for calculation. However, the demand for the product, possible market share, possibilities of compounds purification, and other important market and technological factors have to be considered. In addition, today not all wild plantations are used for industrial berries collection, and the amount of SBT biomass could be approximately 4 times less [63], correspondingly. For more precise market value data, a separate, more profound research is necessary which is not the aim of this article. However, even such rough calculations show that SBT biomass contribution to the market is comparable with berries market value.

The evaluation of the best harvesting time and possibilities for the use of SBT biomass for the pruning of shrubs, as well as the comparison of seasonal changes in the phytochemical profile and biological activity of hydrophilic extracts, will be performed in further research.

## Figures and Tables

**Table 1:** Dominant compounds in the chromatograms of SBT twigs.

					Relative Abundance, %					
Peak No.	t^R^ (min)	[M–H]^−^(m/z)	Fragments	Identification ^1^	Tatiana		Maria Bruvele		Bot. Lub.	
					Water	50% EtOH	Water	50% EtOH	Water	50% EtOH
1	0.41	341	179; 161; 143; 119; 113; 101	Sucrose, fructose, glucose	9.7	9.0	10.7	8.2	10.9	9.3
2	0.47	191	111; 173; 127; 85	Quinic acid	5.7	4.4	6.1	5.0	5.6	4.2
3	0.98	175	159; 147	Serotonin ^2^	0.1	0.1	0.1	0.1	0.1	0.1
4	1.84	305	179; 125	Gallocatechin or its isomer epigallocatechin	0.7	1.8	2.6	1.7	3.2	1.5
5	1.89	593	407; 425; 305; 467; 289	(epi)catechin-(epi)gallocatechin	2.2	0.5	0.9	1.1	1.3	0.4
6	1.97	1185	881; 593; 305; 289; 245	Procyanidin tetramer	4.1	2.2	2.6	1.0	2.8	1.7
7	2.06	1055	881; 593; 305; 289	Procyanidin tetramer	4.4	1.8	4.6	2.7	4.4	2.3
8	2.30	865	577; 289; 245	Procyanidin trimer	4.1	4.5	5.2	4.9	4.2	3.4
9	2.38	289	245; 125	Catechin/Epicatechin	6.9	5.3	6.7	8.1	7.0	4.2
10	2.50	1153	865; 577; 289; 245	Procyanidin tetramer	13.5	13.0	9.6	12.2	17.2	7.0
11	3.51	610	-	Contaminant from solvent—ethanol, nylon filter	0.1	1.4	1.7	0.7	0.1	1.1
12	4.28	723	-	Contaminant from solvent—ethanol, nylon filter	0.6	5.2	7.2	1.3	1.0	5.6
13	5.04	836	-	Contaminant from solvent—ethanol, nylon filter	0.6	4.6	6.5	1.8	1.4	5.2
14	5.77	949	-	Contaminant from solvent—ethanol, nylon filter	0.3	1.1	1.8	0.6	0.4	1.3
15	3.28	609	301; 271	Quercetin-3-O-rutinoside	-	-	-	0.01	-	-
16	3.33	301	286; 109	Quercetin	0.1	0.1	0.2	0.4	0.1	0.1
17	7.14	487	293; 117	Triterpenoid	-	2.8	3.0	2.5	-	3.0
18	7.79	471	452; 265; 117	Triterpenoid	-	1.8	-	3.1	-	2.1
19	7.86	471	265; 117	Triterpenoid	-	1.9	-	1.8	-	2.4
20	8.07	455	277; 117	Triterpenoid	3.2	3.4	7.2	4.3	-	-
21	8.01	617	255; 117	Acylated triterpenoid	-	-	-	-	-	2.8

^1^ Serotonin showed weak signal in negative ion ESI LC-MS, which was quantified in MRM positive ionization mode. ^2^ Compounds were tentatively identified compared with those reported in the literature and confirmed through databases, specifically the Dictionary of Natural Products and ChemSpider, focusing on MS/MS fragmentation patterns and accurate mass.

**Table 2:** The comparison of relative peak areas of dominant biologically active compounds calculated on mg/mg extract.

No.	Tentative Identification	Relative Peak Area (Relative Units)		Cultivar
		50% EtOH	H_2_O	
1	Quinic acid	25,954	31,176	Tatiana
		43,608	53,949	Maria Bruvele
		38,504	44,680	Bot. Lub.
2	Gallocatechin or its isomer epigallocatechin	14,917	11,890	Maria Bruvele
		2369	4476	Tatiana
		5291	8424	Bot. Lub.
3	(epi)catechin-(epi)gallocatechin	6283	2855	Maria Bruvele
		4782	5539	Tatiana
		5314	6759	Bot. Lub.
4	Procyanidin tetramer	8054	3230	Maria Bruvele
		1674	4395	Tatiana
		5335	5841	Bot. Lub.
5	Procyanidin tetramer	8879	2397	Maria Bruvele
		-	4665	Tatiana
		-	7803	Bot. Lub.
6	Procyanidin trimer	37,952	31,036	Maria Bruvele
		15,879	26,104	Tatiana
		19,967	33,381	Bot. Lub.
7	Catechin/Epicatehin	51,282	56,089	Maria Bruvele
		24,115	34,058	Tatiana
		35,098	46,065	Bot. Lub.
8	Procyanidin tetramer	3004	1739	Maria Bruvele
		916	3500	Tatiana
		1864	3656	Bot. Lub.
9	Procyanidin tetramer	9768	5576	Maria Bruvele
		2897	6506	Tatiana
		4632	7877	Bot. Lub.
10	Procyanidin tetramer	635	2850	Maria Bruvele
		236	619	Tatiana
		382	751	Bot. Lub.
11	Procyanidin tetramer	1348	973	Maria Bruvele
		1893	1023	Tatiana
		2946	3040	Bot. Lub.
12	Quercetin-3-O-rutinoside	334	-	Maria Bruvele
		-	-	Tatiana
		-	-	Bot. Lub.
13	Quercetin	2759	677	Maria Bruvele
		933	1003	Tatiana
		1287	3105	Bot. Lub.
14	Triterpenoid	3218	-	Maria Bruvele
		3750	-	Tatiana
		4561	-	Bot. Lub.
15	Triterpenoid	7631	-	Maria Bruvele
		5000	-	Tatiana
		6950	-	Bot. Lub.
16	Triterpenoid	7273	-	Maria Bruvele
		5860	-	Tatiana
		8651	-	Bot. Lub.
17	Triterpenoid	4923	14,713	Maria Bruvele
		8978	6910	Tatiana
		-	-	Bot. Lub.
18	Acylated triterpenoid	-	-	Maria Bruvele
		-	-	Tatiana
		6808	-	Bot. Lub.

**Table 3:** Antimicrobial activity of the extracts from SBT samples.

	Maria Bruvele	Bot. Lub.	Tatiana	Maria Bruvele	Bot. Lub.	Tatiana
	50% EtOH Extract, mg/mL			Water Extract, mg/mL		
*E. coli* MIC/MBC	0.20/0.20	0.39/0.39	0.39/0.39	0.39/0.39	0.78/50	0.39/0.39
*P. aeruginosa* MIC/MBC	0.39/0.78	0.78/1.56	3.13/3.13	0.39/3.13	0.78/50	0.78/1.56
*S. aureus* MIC/MBC	0.20/0.39	0.39/0.78	0.20/0.78	0.39/0.78	0.39/12.2	0.39/0.78
*B. cereus* MIC/MBC	0.39/50	0.78/>50	0.78/50	0.78/>50	0.78/>50	0.78/50
*C. albicans* MIC/MFC	0.20/>50	0.20/>50	0.39/>50	0.20/>50	0.39/>50	0.39/>50
	**Antibiotics**					
	**Gentamicin (Reference), µg/mL**		**Fluconazole (Reference)** **, µg/mL**		**Tetracycline Hydrochloride [32], mg/mL**	
*E. coli* MIC/MBC	1.00/4.00		ND		0.76	
*P. aeruginosa* MIC/MBC	0.25/4.00		ND		ND	
*S. aureus* MIC/MBC	0.25/4.00		ND		1.52	
*B. cereus* MIC/MBC	ND		ND		0.76	
*C. albicans* MIC/MFC	ND		32/>256		ND	

MIC—minimum inhibitory concentration; MBC—minimum bactericidal concentration. MFC—minimum fungicidal concentration; ND—not determined. MIC tests were performed in triplicate for each strain and antimicrobial compound. Confidence interval is ±0.01 at α = 0.05.

## Data Availability

Data are contained within the article.
